# Life-history traits of the spiny dogfish *Squalus acanthias* in the Adriatic Sea

**DOI:** 10.1038/s41598-019-50883-w

**Published:** 2019-10-04

**Authors:** Giada Bargione, Fortunata Donato, Mario La Mesa, Carlotta Mazzoldi, Emilio Riginella, Claudio Vasapollo, Massimo Virgili, Alessandro Lucchetti

**Affiliations:** 1 National Research Council (CNR), Institute for Biological Resources and Marine Biotechnologies (IRBIM) of Ancona (Italy), Largo Fiera della Pesca, 1, 60125 Ancona, Italy; 2 University of Padova, Department of Biology, Via U. Bassi 58/B, 35131 Padova, Italy; 3 Zoological Station Anton Dohrn, Villa Comunale, 80121 Naples, Italy

**Keywords:** Conservation biology, Ichthyology

## Abstract

Pivotal life history traits concerning age structure and reproduction of the spiny dogfish (*Squalus acanthias*, Linnaeus 1758) were investigated in the Adriatic Sea from mid February 2012 to mid July 2013 and in 2016. The whole sample consisted of 176 females and 150 males, ranging between 217–1025 mm and 219–875 mm, respectively. The individual age, which was estimated using a cross-sectioning technique of the second dorsal-fin spine, ranged from 0 to 13+ years for females and from 0 to 9+ years for males. Based on the length-at-age estimates, the Gompertz growth parameters were L_∞_ = 1130 mm, k = 0.18 and L_∞_ = 920 mm, k = 0.24 for females and males, respectively. The size at sexual maturity (L_50_) was 659 mm for females and 575 mm for males, corresponding to 7.5 and 5.5 years of age (A_50_), respectively. Mean biennial fecundity was approximately 11 embryos/female and 12 ripe oocytes/female. Mature males occurred during much of the sampling period, while mature females with nearly full-term embryos were exclusively recorded in May 2013 and July 2016. Monitoring of catches conducted in a sample port of the north Adriatic (Chioggia) over the past 20 years has shown fluctuating trends in landings, with peaks during the summer reproductive season.

## Introduction

The Mediterranean Sea offers a unique perspective on fish population declines over historical timescales, as the resources have been highly exploited since a long time^1–3^. Elasmobranchs are highly vulnerable to overfishing due to their life history characteristics that include long life span, late sexual maturity, large size at birth and low reproductive rates^4^. Fishery removes selectively larger individuals, causing a general decline in size. Considering the positive relationships between fecundity and female size, this causes a reduction in the reproductive potential (REF) and in the number of possible mating events per female^5^. Until now information on the status of population are missing and this did not allow developing sharks conservation measures in the Mediterranean Sea^2^.

Life history traits awareness of overfished species is fundamental to understand the effects of overexploitation on the conservation of the species and to implement sustainable management strategies^6^. Critical key factors as size and age frequency distribution and fecundity, in relation- to female size and size/age of sexual maturity are, therefore, of priority importance to assess the population structure, dynamics and the reproductive potential of exploited stocks^7,8^.

The shallow northern-central part of the Adriatic Sea represents one of the most suitable fishing grounds of mixed multi-specific demersal fisheries in the Mediterranean, which have already caused a decrease in elasmobranchs diversity and abundance (Jukic-Peladic *et al*.^9^; Ferretti *et al*.^10^; Barausse *et al*.^11^). In the Adriatic Sea, accessory catches of many species of cartilagineous fish include *Squalus acanthias*, *Mustelus mustelus* and *M. punctualtus*, *Raja asterias, R. clavata, R. miraletus*, *Torpedo spp*., *Scyliorhinus canicula, S. stellaris*, *Galeorhinus galeus*, *Alopias vulpinus* and juveniles of *Carcharhinus plumbeus*^11,12^. Among them, the spiny dogfish (*S. acanthias*) is one of the most important valuable commercial species commonly caught by bottom^9,11,13^ and midwater pelagic trawl^11,14,15^ and passive nets^16,17^.

Currently, *S. acanthias* is globally listed by the IUCN as vulnerable, whereas in the Mediterranean Sea it is classified as endangered^18^. The spiny dogfish is a highly migratory small demersal shark, with a worldwide distribution except for the tropical and polar regions^19^, and the north Pacific, where genetic analyses revealed that *Squalus suckleyi* replaces *S. acanthias*^20^. As many elasmobranchs, *S. acanthias* is a K-selected species with slow growth rates, low fecundity and late sexual maturation^21,22^, and tend to aggregate by sex and size^19,22^. The spiny dogfish is an aplacental viviparous species^22,23^, with a long gestation, estimated up to two years^24–26^.

The main aim of this study is to investigate some key life-history traits of the spiny dogfish in the northern-central Adriatic Sea, an area where this species is frequently caught as bycatch. Basic biological characteristics such as age, growth, length-mass relationship, size frequency distributions, size and age at sexual maturity and fecundity were investigated to improve current knowledge of this species in this area. Furthermore, based on official landings of the spiny dogfish of the major fishing harbor of the Northern Adriatic Sea (Chioggia), we provided information on temporal trend of catches in the last two decades to infer the status of the Adriatic spiny dogfish population.

## Materials and Methods

### Sample collection and processing

**Figure 1:**
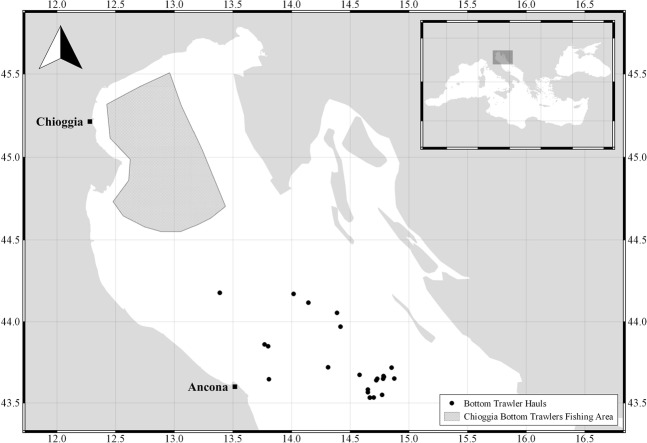
Map of Chioggia bottom trawler fishing area and the geographic point of spiny dogfish caught by trawlers of Ancona harbor.

The spiny dogfish were collected monthly off Chioggia and Ancona harbours, which are located in two different areas of the Adriatic Sea (Fig. 1). A total of 223 samples were collected from landings at the Chioggia harbour between February 2012 and July 2013, while 103 were taken aboard a bottom trawler operating in the Ancona harbour between February and December 2016. The bottom trawler catches occurred on sandy bottoms ranging from 40 to 90 m depth at 15–50 nm far from the coast. All the specimens sampled were purchased from commercial suppliers and no live fish were handled in the study. The relative percentage of males and females (sex ratio) was determined on the whole sample. Departure from the expected 1:1 sex ratio was tested for the sampled population using a χ^2^ goodness-of-fit test.

All specimens were frozen at −20 °C until dissection in the laboratory. Each specimen was measured to the nearest mm from the tip of the snout to the tip of the upper lobe of the caudal fin (total length, TL) and weighed to the nearest gram (total mass, TM). Sex was established by recording presence/absence of claspers.

### Reproduction

The abdomen of each individual was cut with a knife from anus to throat, and the gonads were removed and weighed. Gonads were macroscopically examined to determine the maturity stage for males and females, following the description provided by the Report of the Workshop on Sexual Maturity Staging of Elasmobranchs^27^.

In 150 males, sexual maturity was determined by clasper length and calcification (Conrath, 2005), testes shape and amount of sperm stored in the testes/epididymis/sperm sac. Five maturity stages were considered: M1 (immature), M2 (developing), M3a (spawning capable), M3b (actively spawning), M4 (regressing). At the Chioggia harbour, before the specimens being sold, 407 additional males beyond the 150 processed in the laboratories were staged by direct examination of claspers length and calcification; those with flaccid claspers shorter than pelvic fins were classified as immature, while the others with rigid claspers longer than pelvic fins as mature.

In 176 females, sexual maturity was determined from the size of ova and the state of uteri. Seven maturity stages were considered: F1 (immature), F2 (developing), F3a (capable to reproduce), F3b (early pregnancy), F3c (mid pregnancy), F3d (late pregnancy), F4a (regressing), F4b (regenerating).

Size at sexual maturity (TL_50_) was assessed for both sexes by fitting a logistic model to the proportion of mature specimens (i.e. at stages 3–4) per size class.

The mean gonadosomatic index (GSI_mean_), which is the percentage of gonad mass (GM) to total body mass (TM), was calculated for each maturity stage both for males and females, in which it was possible to extract and weight the gonads, as a measure of reproductive investment.

In mature females, both ovarian and uterine fecundity were assessed. Uterine fecundity was estimated as the number of embryos in the uteri, whereas ovarian fecundity was assessed as the number of ripe oocytes in the ovaries. Each embryo was weighed, measured and sexed (when possible). A linear regression was used to determine the relationship between fecundity and size of mature females.

### Age and growth determination

For age determination, the second dorsal fin spine was removed by cutting horizontally just above the vertebrae. Spine cross-sections were obtained following the procedure described by Soldat^28^. For each spine, the number of annuli was determined by counting the alternating translucent and opaque zones visible on the spine cross-section^29^ using a light microscope (Leica DM4000B) at 4x magnification. Assuming that the annuli were laid down yearly, the age of fish was estimated by counting all the translucent zones. The final individual age was then calculated in months, based on the date of capture and presumed common birthday (June). Two readings were performed by two different readers one week apart. The precision of age readings was estimated by calculating the average percentage error (APE) and the coefficient of variation (CV)^30,31^.

To describe the growth pattern, the von Bertalanffy and the Gompertz growth models were initially fitted to the length-at-age data estimated for each sex. Growth parameters and 95% confidence intervals were estimated by applying a non-linear regression analysis, and the Akaike Information Criterion (AIC)^32^ was calculated for the best model selection. Statistical analyses were performed using the Growth II software (PISCES Conservation Ltd, Lymington, UK).

Age of maturity was assessed both for females and males from the resulting growth-curve knowing the size of maturity (TL_50_).

The length-mass relationship was estimated for each sex using the function TM = aTL^b^, where TM is the total body mass (g) and TL is the total length (mm). Isometric growth (i.e. b = 3) departure was tested using a t-Student test. The allometric indices (b) calculated for males and females were compared by applying an F test.

### Fishery data

Fishery data include official landing statistics of the Chioggia harbor^33^, which hosts the major fleet of the northern Adriatic Sea^34^. Total commercial landings, expressed in biomass of landed specimens, of spiny dogfish were analyzed for the last two decades (1997–2016), since before 1997 the spiny dogfish was registered with other shark species (Barausse *et al*.^11^). Total fishing capacity (GRT, gross registered tonnage) of the Chioggia trawlers was used as a proxy of fishing effort, because estimates of fishing effort were not available. Therefore, annual CPUEs were estimated by dividing total landings (*t*) per total fishing capacity.

Mean monthly landings across years were also calculated to investigate monthly tons variations.

### Compliance with ethical standards

All applicable international, national, and/or institutional guidelines for the care and use of animals were followed. All specimens collected were died at the time of sampling.

## Result

### Sample data

**Figure 2:**
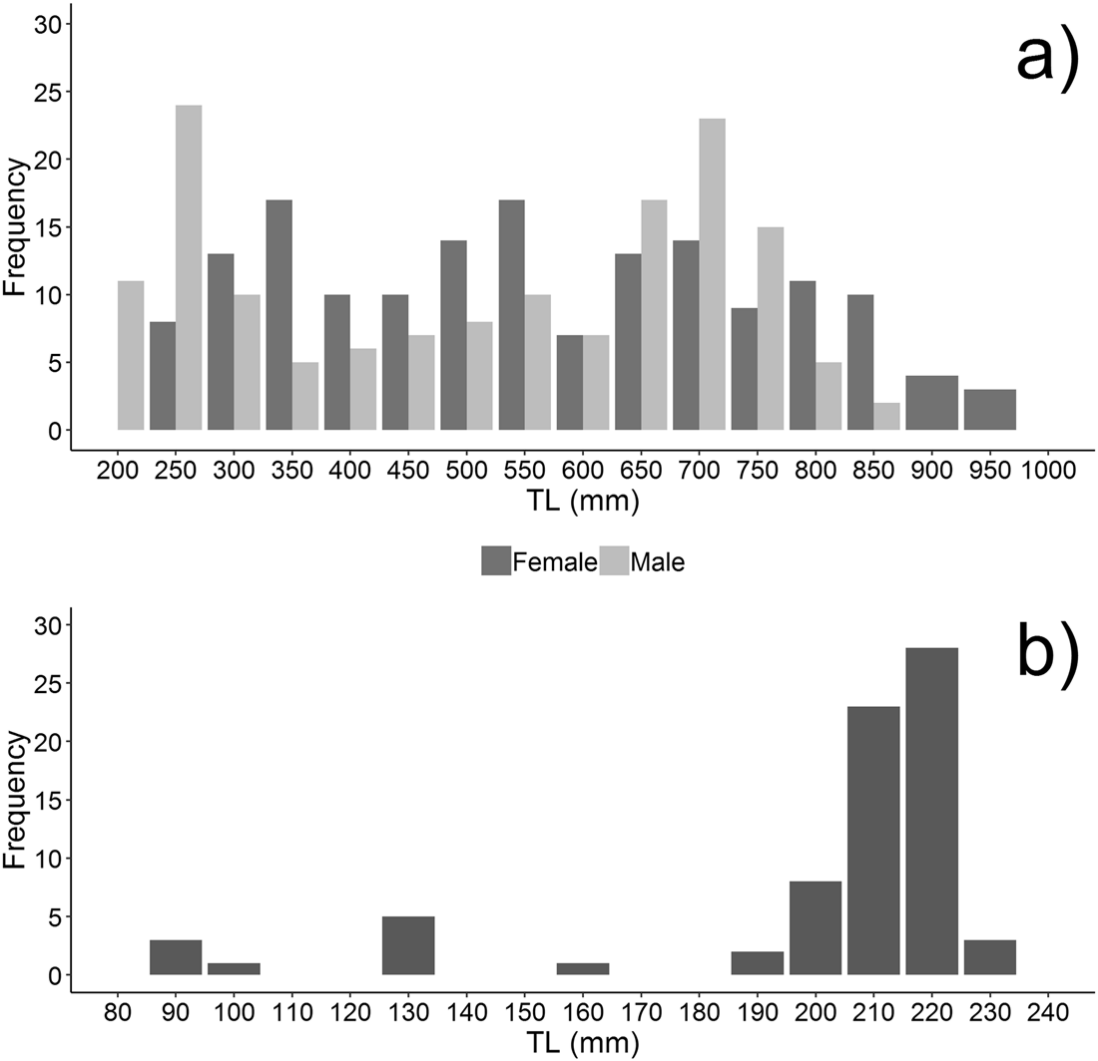
Size frequency distribution for both sexes (**a**) and embryos (**b**).

Overall 176 females and 150 males of the spiny dogfish were collected during the sampling period. Size ranged from 217 to 1025 mm (mean ± SD, 577 ± 216 mm) for females and from 219 to 875 mm (mean ± SD, 533 ± 201 mm) for males (Fig. 2). Females apparently exhibited wider size range and higher mean total length but the Kolmogorov-Smirnov test did not show any significant difference in length frequency distribution between sexes (d = 0.146; p = 0.15). A total of 74 embryos ranging from 81 to 228 mm, with a modal birth size of 210–220 mm, were found in seven pregnant females, and it was possible to sex 47 of them (28 females and 19 males) (Fig. 2). Excluding embryos, the sex ratio of the sampled population of spiny dogfish was not significantly different from a 1:1 (*χ*^2^ = 2.07 test, P = 0.15).

### Age and growth

**Figure 3:**
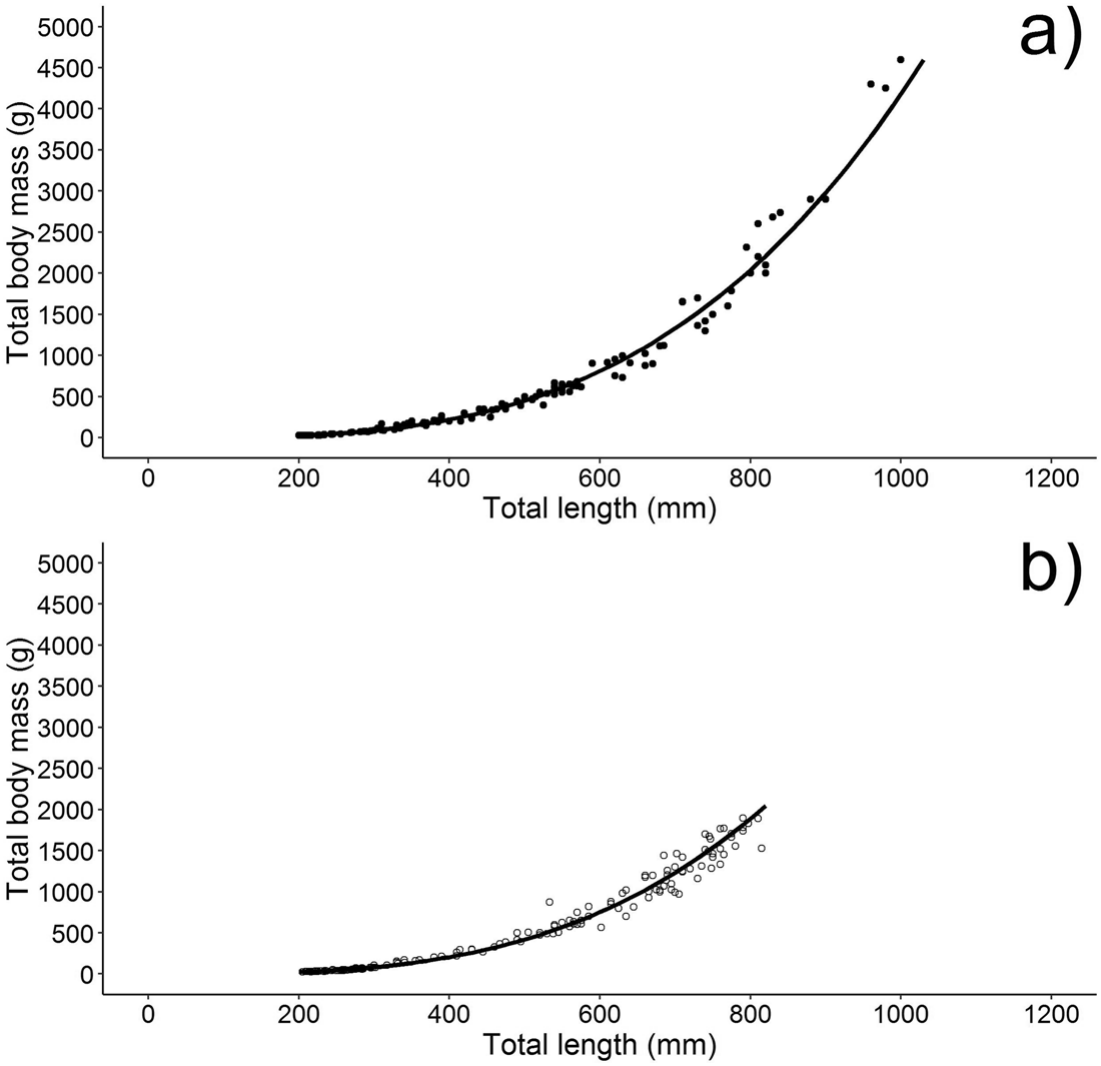
Length-mass relationship for females (**a**) and males (**b**).

A positive relationship was found between fish size and mass (Fig. 3), as summarized in the following equations for females and males, respectively:

$$\begin{array}{lll}{\rm{TW}}=9.50\times {10}^{-7}{{\rm{TL}}}^{3.21} & {\rm{n}}=176 & {{\rm{r}}}^{2}=0.99\\ {\rm{TW}}=9.54\times {10}^{-7}{{\rm{TL}}}^{3.20} & {\rm{n}}=150 & {{\rm{r}}}^{2}=0.99\end{array}$$

The b-values were significantly higher than 3 in both sexes, indicating a positive allometric growth (t-test, df = 1, P < 0.05), with no difference between them (F_(1,322)_ = 0.00022, P = 0.98).

**Figure 4:**
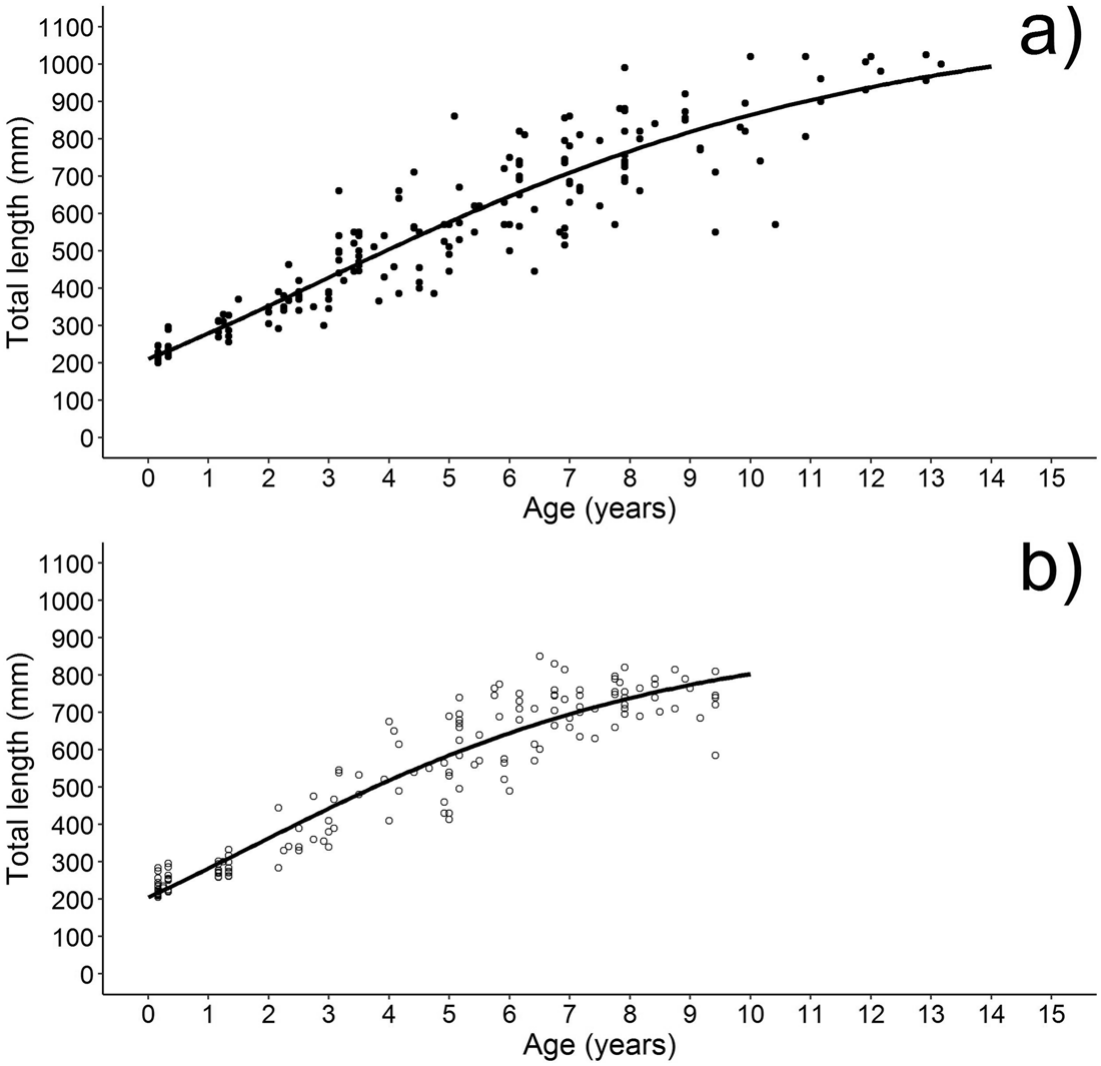
Gompertz growth model fitted to the length-at-age data estimated for females (**a**) and males (**b**) of spiny dogfish from the Adriatic Sea.

Age was successfully determined for all the specimens caught. The estimated age range was between 0 and 13+ years and between 0 and 9+ years for females and males, respectively (Fig. 4). Based on the AIC scores, the Gompertz, TL = L_∞_ exp(−exp(−k(t − l))), where L_∞_ is the asymptotic length, k is the growth rate, t is the age and l is the age at the inflection point, was the best model describing the spiny dogfish body growth. The theoretical asymptotic length (L_∞_) were 920 mm (95% CI = 815–1024) and 1130 mm (95% CI = 986–1273) for males and females, with an instantaneous growth rate (k) of 0.24 year^−1^ (95% CI = 0.19–0.29) and 0.18 year^−1^ (95% CI = 0.14–0.22), respectively. Indices of age precision between readers and within readings of the same reader were both relatively low (APE = 5.7% and 5.2%; CV = 8.0% and 7.3%), indicating good consistency (or reproducibility) of age estimates.

### Reproduction

**Figure 5:**
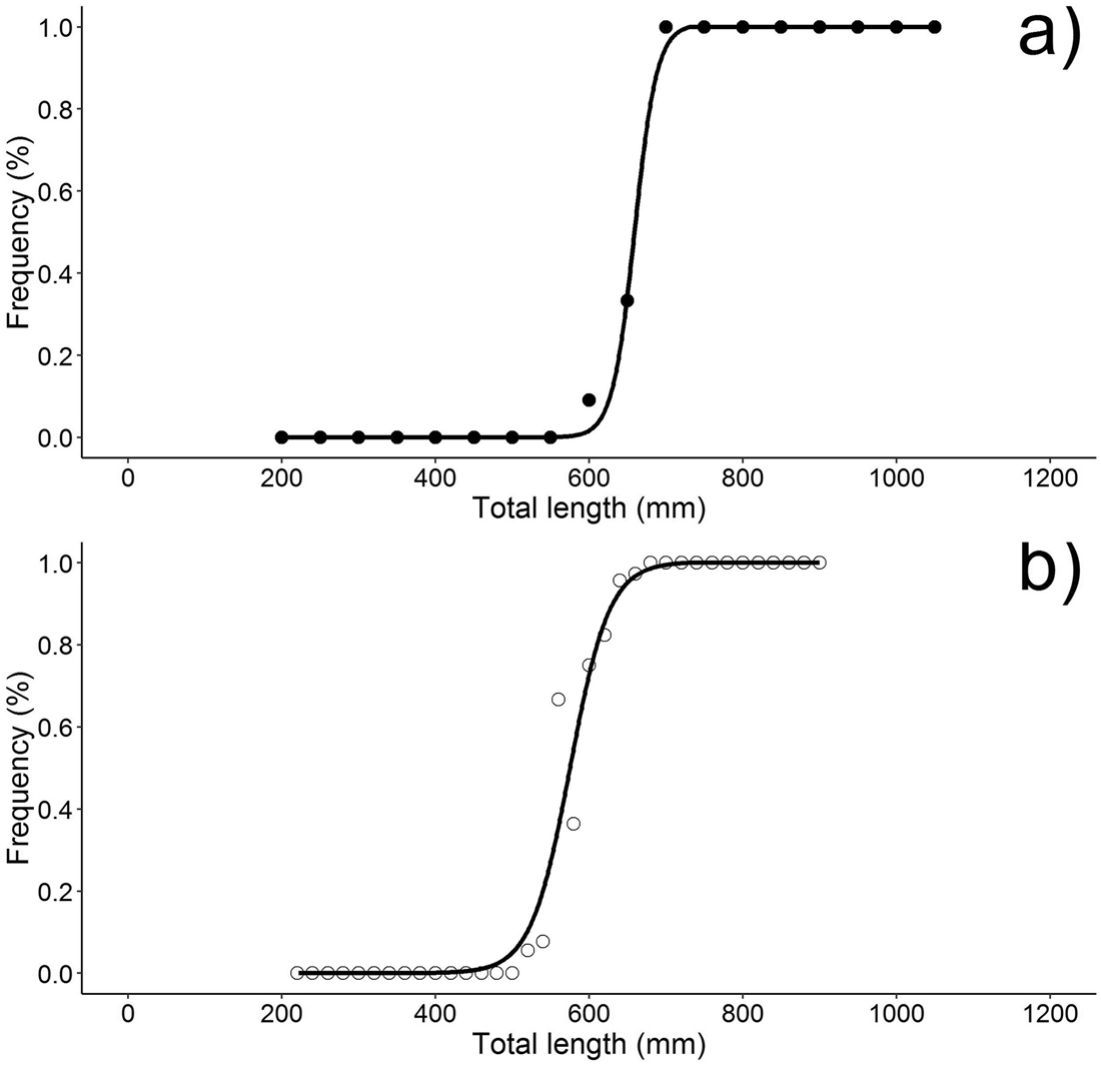
Size at sexual maturity (L_50_) assessed for females (**a**) and males (**b**) of spiny dogfish from the Adriatic Sea.

Fish size at sexual maturity (TL_50_) was assessed for 557 males (additional males being sampled at Chioggia by direct examination of clasper morphology, see methods) and 157 females. Size at sexual maturity was different in males and females (Fig. 5), being respectively 575 mm (95% CI = ± 8.7) and 659 mm (95% CI = ± 2.4), which roughly corresponded to an age of 5.5 and 7.5 years respectively.

The gonadosomatic index (GSI) was calculated for 18 females and 37 males, from which it was possible to remove gonads at different maturity stages. GSI values consistently increased with gonad maturation, being 0.2%, 1.5%, 1.6% and 1.7% in maturity stages M1, M2, M3a and M3b, and 0.3%, 0.5%, 3.0%, 6.4%, 5.1% and 13.3% in stages F1, F2, F3a, F3b, F3c and F3d, respectively.

**Figure 6:**
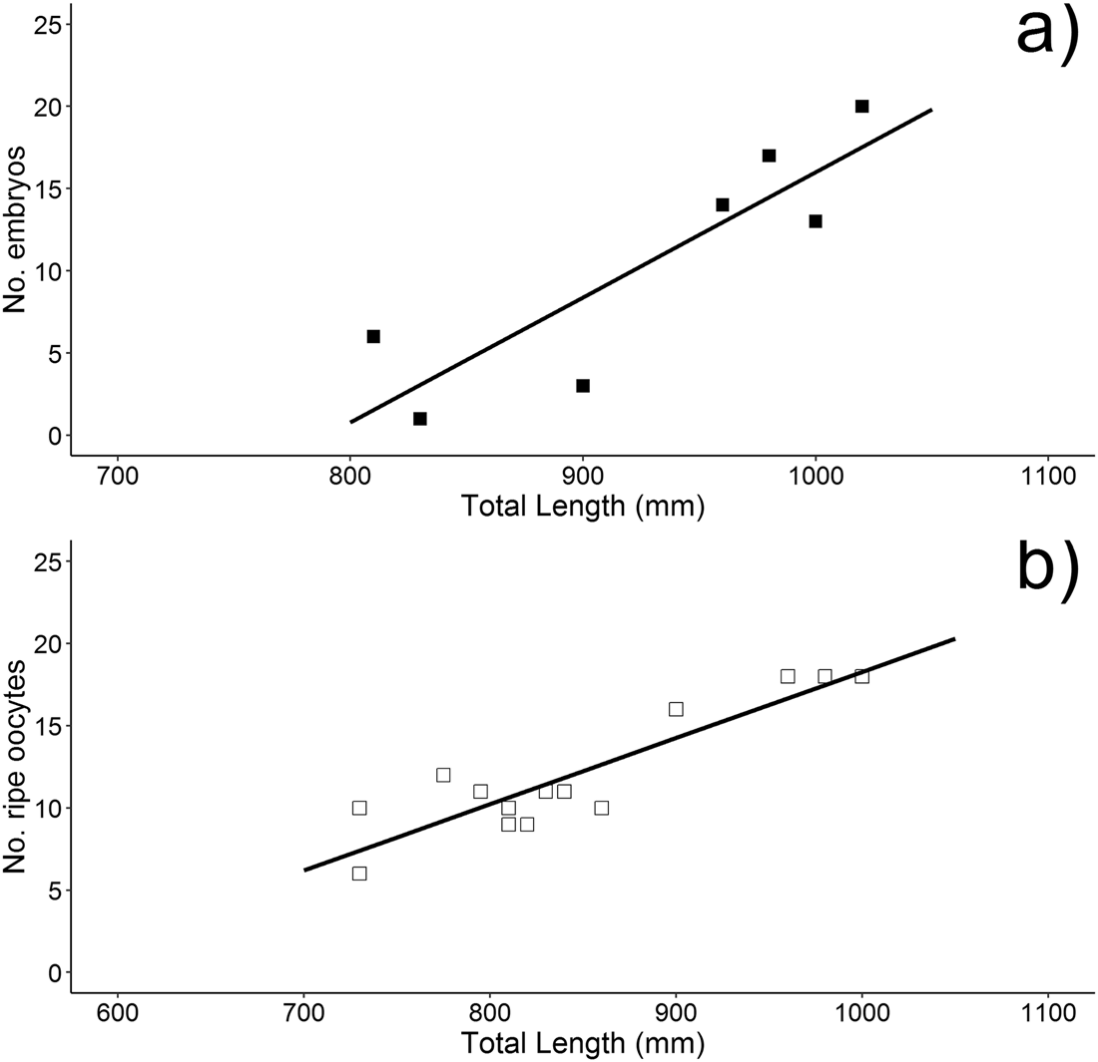
Relationships between female size and fecundity as number of embryos (**a**) and number of ripe oocyte (**b**) of spiny dogfish from the Adriatic Sea.

During the sampling period, pregnant females with nearly full-term embryos (yolk sac almost completely resorbed) were exclusively found in May 2013 and July 2016, whereas mature males with sperm coming out at pressure were present all the year round. Ovarian fecundity, based on the number of ripe oocytes ranging from 15 to 40 mm (mean ± SD, 26.59 ± 8.13 mm), was counted in both ovaries of 14 mature females, and ranged from 6 to 18, with a mean of 12 ± 3.85 ripe oocytes/female. It showed a positive relationship with size of mature females, as summarized in the following equation (Fig. 6):

$${{\rm{F}}}_{{\rm{ovarian}}}=-\,21.96+0.04(95 \% \,{\rm{CI}}=0.03-0.05)\,{\rm{TL}}\,(n=14,\,{{\rm{r}}}^{2}=0.80).$$

Uterine fecundity, calculated as number of embryos in both uteri of 7 pregnant females, ranged from 1 to 20, with a mean of 10.6 ± 7.27 embryos/female. Once again, it showed a positive relationship with size of females, as summarized below (Fig. 6):

$${{\rm{F}}}_{{\rm{uterine}}}=-\,60.17+0.07\,(95 \% \,{\rm{CI}}=0.02-0.12)\,{\rm{TL}}\,(n=7\,{{\rm{r}}}^{2}=0.76)$$

### Fishery data

**Figure 7:**
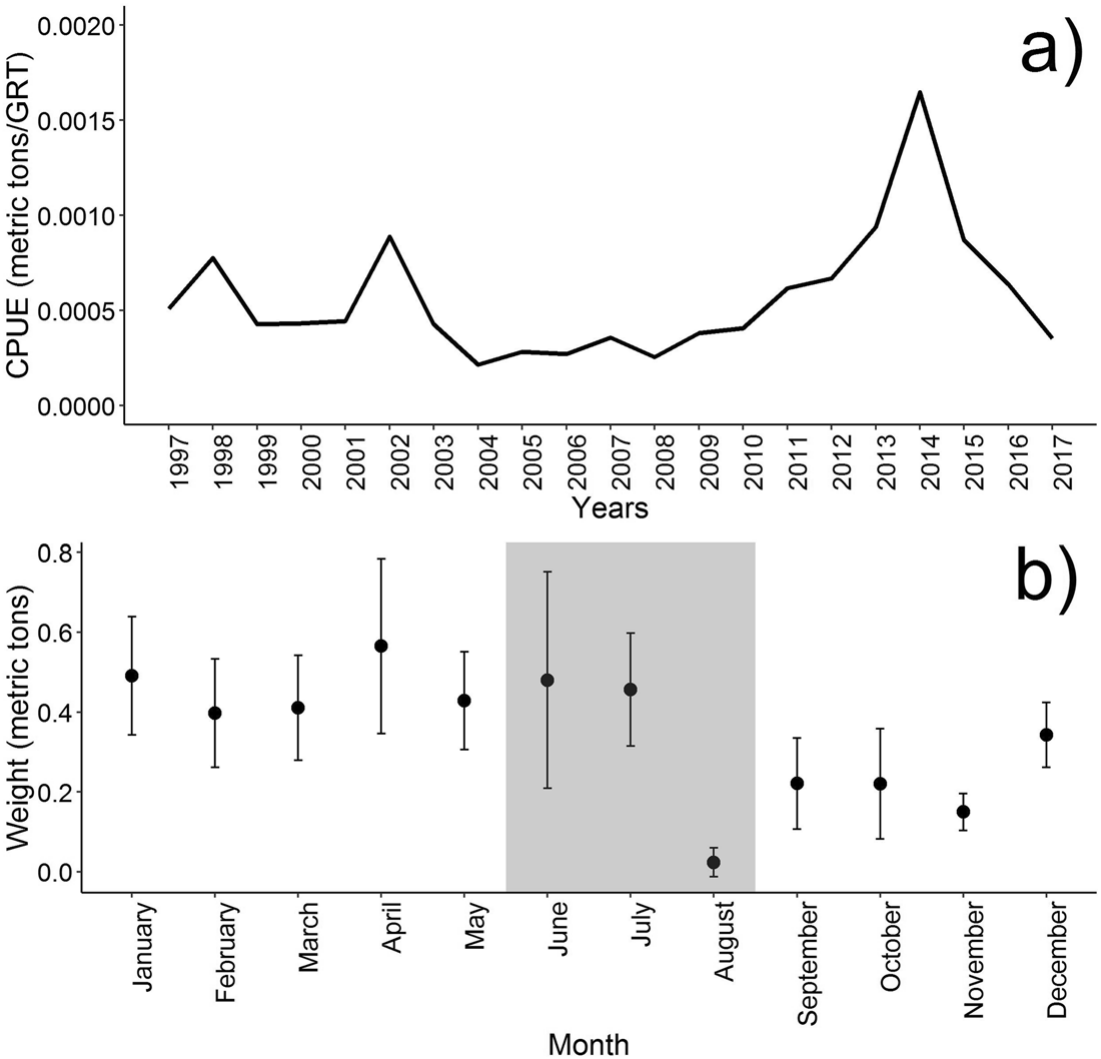
Spiny dogfish CPUE in the last two decades (**a**) and monthly landings of spiny dogfish showing the reproductive period of the species (grey area) (**b**); bars indicate 95% confidence interval.

Based on fish landings in the Chioggia harbour, the catch per unit of effort (CPUE) showed a fluctuating trend in the last two decades. Three main peaks were observed in 1998, 2002 and 2014, each followed by a steep decline afterwards. Mean monthly landings (tons) across years showed three main peaks in January, April and June-July, just before the closure of fishing activities in August in the whole area (Fig. 7).

## Discussion

The present study reports key information on the life history traits of spiny dogfish in the northern-central Adriatic Sea, focusing the attention on age determination and reproductive biology. The growth bands formed on the external surface of the second dorsal-fin spine have been generally used to estimate individual age of this species, both in the Mediterranean Sea (e.g. Gračan *et al*.^35^) and elsewhere (e.g.^29,36,37^. However, according to Holden and Meadows^29^, the use of external growth bands as ageing method is error prone, due to some inherent difficulties linked to their morphology and/or interpretation (e.g. worn spines, basal erosion of the enamel, false rings). A more recent study indicated that age estimates based on external growth bands were overestimated compared to those obtained by sectioned vertebrae^37^. As a results, a pletora of different growth curves and longevity estimates has been reported for this species, only partially due to different growth models used or different sampling areas (e.g. Tribuzio *et al*.^38^).

**Figure 8:**
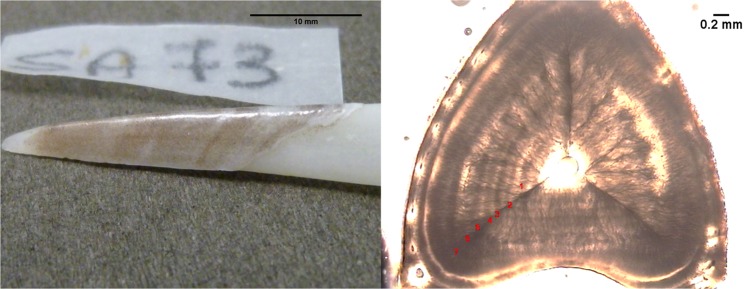
Whole (left) and cross-section (right) of second dorsal-fin spine of a spiny dogfish (male, 765 mm,1450 g) aged 7 years old.

In this study, the individual age of the spiny dogfish was estimated using the second dorsal-fin sectioning procedure^28^, as the external growth bands observed in our samples exhibited an irregular and rather confuse patterns (see Fig. 8). Following this methodology, the maximum age estimated for males and females was 9+ and 13+ years, respectively. A recent study reported age estimates for spiny dogfish sampled in the same area (Adriatic Sea), using the external growth bands to determine individual age of fishes^35^. Despite the same fish size range sampled, the maximum age estimated for males and females by Gračan *et al*.^35^ was considerably higher than our estimates, being 23 and 36 years, respectively. As a consequence, the growth parameters and age at sexual maturity calculated from the length-at-age data were not consistent from each other as well.

The wide sampling area of the northern-central Adriatic Sea covered by fishing fleets operating in Chioggia and Ancona harbour, and the relatively long sampling period spanning almost three years, make us confident that the fish size range sampled was representative of the spiny dogfish really present in the basin. Therefore, the maximum age estimated in this study likely represented the life span of this species in the Adriatic Sea. On the other hand, the long life span and the late sexual maturity reported by Gračan *et al*.^35^ can be hardly claimed considering the huge fishing effort in the Adriatic Sea^39^ and the low selectivity of Mediterranean bottom trawl^40,41^. Differences in size at sexual maturity and the duration of the life span are frequently attributed to latitudinal variability of biological characteristics in chondrichthyans^42,43^. For example in the Atlantic Ocean for the spiny dogfish a longer life span (17 and 24 years for males and females, respectively)^37^ and a bigger size and age at maturity (720 mm and 14 years for males, 940 mm and 36 years for females)^44,45^ have been reported. The lack of significant sex ratio bias commonly recorded from different areas indicates that spiny dogfish are generally characterized by balanced populations (Avsar^46^; Demirhan, S. A., Seyhan^47^; present study).

Data on individual maturity stages and relevant GSI enabled us to describe the reproductive cycle of spiny dogfish in the Adriatic Sea. The GSI of immature specimens was comparable between sexes, accounting for less than 0.5% of total body mass. At sexual maturity, GSI of males increased until reaching approximately 2% of total body mass, remaining roughly the same across the mature gonad stages. Conversely, GSI of females at sexual maturity exhibited a steady increase, attaining for more than 13% of total body mass in pregnant females with fully formed embryos. Other authors inside (i.g. Chatzispyrou and Megalofonou^48^; Capapé and Reynaud^49^) and outside (i.g. Demirhan and Seyhan^50^; Cahide and Ismen^36^) the Mediterranean Sea analyzed GSI related to size finding GSI values higher for females especially during advanced gestation stages. Females dogfish seem to invest a lot of energy to produce gonadic products as they attain maturity.

The spiny dogfish is an ovoviviparus species with a commonly reported very long gestation period, lasting between 18 to 24 months depending on sampling areas^19^. Braccini *et al*.^51^ reported that during pregnancy, both embryos and unfertilized oocytes develop inside the belly of pregnant specimens so that, soon after puppies birth, females already have ripe oocytes and are ready to reproduce again. In the Adriatic Sea, we recorded pregnant females carrying nearly full-term embryos with small yolk sacs only in summer, while mature males with sperm coming out at pressure were almost found all the year round. Sex related difference in reproductive strategies in this species was already observed in the same area, where mature females were caught exclusively in summer and mature males in June, November and December^52^. For *S. acanthias* is reported a short parturition-fertilization interval as, as soon after parturition, the female ovulate and conceive again^49^. Sperm storage has been reported in other elasmobranchs storing sperm in the oviducal glands^53^. Nevertheless, the oviducal glands of spiny dogfish have a simple structure, enabling sperm storage for a reduced time^54^. Indeed, considering the synchronism between females and males during the summer season, found in our study and in the previous one conducted always in the Adriatic Sea, it suggests that sperm retention in females spiny dogfish will not be necessary for long time.

Both ovarian and uterine fecundity resulted to be positively correlated with females size, as larger females have commonly more space to bear pups^49,55^. As a common feature in the spiny dogfish^56^, ovarian fecundity was higher than uterine fecundity, likely due to resorption of unovulated mature eggs or abortion of embryos. The size range of embryos found in the Adriatic Sea fall within those reported from other areas in the Mediterranean Sea (e.g. Chatzispyrou and Megalofonou^48^; Capapé and Reynaud^49^).

As commonly found in elasmobranchs^57^, size and age at sexual maturity was different between sexes of spiny dogfish, females maturing later and at a bigger size than males. Size at sexual maturity reported for this species was rather different among different localities within and outside the Mediterranean Sea (Table 1) which could be attributed to a shared reproductive plasticity of elasmobranchs, driven by different local environmental conditions such as sea water temperature and prey availability, as well as to genetic and physiological factors^42,43^.

**Table 1:** Size at first maturity (L50) estimated for spiny dogfish from different geographic areas.

Area	L50		Source
	Males	Females	
North-central Adriatic Sea	575	659	this study
Adriatic Sea	504	725	^52^
British waters	600	720	^58^
South Africa	490	790	^59^
North-western Atlantic	590	780	^26^
South-eastern Black Sea	820	880	^46^
Western Ireland	550–620	700–880	^60^
Eastern Mediterranean	501–550	505–690	^48^
Sweden	>540	770–840	^56^
Northern Mediterranean Sea	635–700	860–880	^49^
Pacific Ocean	—	930	^24^
North-western Atlantic	—	800	^61^
British waters	—	820	^62^
New Zeland	580	730	^63^
North Sea	—	700	^64^
North-western Atlantic	640	—	^65^
North-eastern Atlantic	590–600	—	^66^
North Aegean Sea	528	564	^36^
Oslofjord	—	810	^25^
British Columbia	720	935	^44^
Strait of Georgia	—	940	^45^

As far as fishery data are concerned, the catch per unit of effort of the spiny dogfish at Chioggia harbour in the last two decades showed high variability. The time to recover the resource between the first two peaks seemed to be much shorter than the time required between the last two peaks. Considering the low reproductive potential of the species, longer recovery time between the second and the third peak might be due to high quantities (tons) of mature specimens caught in the previous years. The complete recovery of the resource at initial levels took almost ten years, which is roughly the period necessary to reach sexual maturity for females (7.5 years) plus the gestation (2 years). Difference in mean tons monthly landed across years showed 3 different peaks, may be due to different fishing areas exploited during the year and to the migratory behavior of the species during the reproductive season and different growth stages.

## Conclusions

Taking into account the shallow waters of the northern-central Adriatic Sea continental shelf (maximum depth less than 50 m) consisting of sandy-muddy sea bed entirely accessible to towing gears and the low reproductive potential of spiny dogfish in terms of gestation time and individual fecundity, the risk that fishing may reduce the stock of breeding animals below the limit beyond which the stock is no longer able to recover is real and cannot be underestimated. As a consequence, the reduction of spiny dogfish bycatch is an urgent matter and mitigation measures should be promptly adopted, fishermen should be trained on releasing live sharks, nursery/mating areas should be identified and closed during specific times of the year. Finally, awareness campaigns should be launched to reduce shark meat consumption, mainly considering that Italy is globally one of the most important importer of it.

## Data Availability

The datasets generated during and/or analyzed during the current study are available from the corresponding author upon reasonable request.
